# Toward accessible quantification of bone mineral density: feasibility of radiographic bone aluminum equivalence (RBAE) determined by digital radiography against DEXA and CT in translational sheep model

**DOI:** 10.1186/s12880-026-02675-8

**Published:** 2026-08-19

**Authors:** Muhammad M. Kamal, Ahmed I. Abdelgalil, Elham A. Hassan

**Affiliations:** 1 Department of Surgery, Anesthesiology and Radiology, Faculty of Veterinary Medicine, Cairo University, Giza, 12211 Egypt; 2 Surgery and Theriogenology Department, Faculty of Veterinary Medicine, King Salman International University, Ras Sidr, 46612 Egypt

**Keywords:** Bone density, Computed tomography, DEXA, RBAE, Digital radiography, Quantitative radiography, Preclinical imaging

## Abstract

**Background:**

The study aimed to explore an accessible quantification of bone mineral density (BMD) through Radiographic Bone Aluminium Equivalence (RBAE) as determined by Digital Radiography (DR), compared to dual-energy X-ray absorptiometry (DEXA) and computed tomography (CT) in ex vivo animal model.

**Methods:**

Metacarpal and metatarsal bones (*n* = 24) from skeletally mature sheep were imaged using standard DR, DEXA, and CT protocols. DR-derived BMD values were calculated from calibrated greyscale intensities using an aluminum step-wedge and image-analysis workflow. Correlation and regression analyses were performed to evaluate the relationship between the different modalities, whereas the intraclass correlation coefficient (ICC) and Bland–Altman analyses were used to assess agreement between the measurement methods.

**Results:**

DR-derived BMD as expressed as BRAE demonstrated strong linear correlation with DEXA (*r* = 0.779, *p* = 0.001) and moderate linear correlation with CT (*r* = 0.536, *p* = 0.04). Across all imaging modalities, metatarsal bones exhibited significantly higher BMD than metacarpal bones, with clear bilateral symmetry observed within each bone type. The consistency of results across modalities strengthens the validity of the findings and supports the reliability of each technique for detecting biologically meaningful differences in BMD. The agreement between DEXA, CT and digital radiography underscores the feasibility of using radiographic BMD to quantify BMD.

**Conclusion:**

Digital radiography, supported by standardized calibration and analysis, yields a reproducible BMD estimates that align closely with DEXA and CT measurements. These findings highlight DR as a cost-effective and accessible option for BMD assessment, while providing valuable baseline insight into normal mineral distribution. This information supports future research on pathological bone loss, imaging validation, and therapeutic strategies to improve bone quality.

## Background

Bone is a dynamic, metabolically active tissue that provides structural support, protects vital organs, and serves as the body’s primary reservoir for calcium [1, 2]. Bone mineral density (BMD), the ratio of bone mass to area, is a critical indicator of the balance between bone formation and resorption. BMD is a determinant of bone structural integrity, skeletal and mechanical bone strength, fracture risk and capacity to withstand physiological loading [3]. It is essential for sequential monitoring of bone remodeling, the outcomes following surgical or regenerative interventions. Quantification of BMD is of the utmost importance in evaluating osteopenic changes characterized by diminished bone mass, deterioration of bone microstructure, and increased bone fragility, significantly impacting the quality of life and imposing a substantial economic burden [4, 5].

In clinical orthopedics and experimental research, several techniques have been developed to noninvasively measure bone mineralization at various skeletal sites. These include radioabsorptiometry (RA), single photon absorptiometry (SPA), dual photon absorptiometry (DPA), quantitative ultrasound (QUS), dual-energy x-ray absorptiometry (DEXA), quantitative computed tomography (QCT) and peripheral QCT (pQCT) [6]. Among these, DEXA is considered the gold-standard modalities for BMD assessment. DEXA provides highly reproducible real BMD measurements, while CT enables three-dimensional evaluation and volumetric BMD analysis with excellent spatial resolution [7, 8]. Although accurate, both methods require specialized equipment, controlled acquisition settings, and are associated with higher operational costs, limiting their availability in clinical practice and diagnostic settings [9]. Therefore, a strong rationale for developing cost-effective, accessible alternative to DEXA and CT imaging for reliable quantification of BMD, particularly in settings where advanced imaging technology is limited or financially prohibitive.

Radiographic examination is widely available, and simple to perform diagnostic modality [10]. Radiography may provide a practical and economically feasible alternative for assessing bone characteristics, providing valuable quantitative insights in situations where advanced imaging modalities are inaccessible or cost-prohibitive. Previous studies have suggested the use of calibrated radiographic greyscale values using an aluminium step wedge with radiographic bone aluminum equivalence (RBAE) to provide an alternative estimate of mineral density using conventional radiography [3, 11]. However, conventional radiography may be highly sensitive to technical factors, exposure settings, beam angle, and poor precision associated with film processing [12]. Digital radiography has gained widespread advance in digital image acquisition and processing. To date, limited promising studies has evaluated the validity of DR for quantifying BMD in animal models [13].

The use of animal models is widely accepted to study bone density and quality, healing dynamics, and orthopedic implant performance [13–15]. Ovine long bones, particularly the metacarpal and metatarsal bones, serve as well-established large-animal model due to their anatomical size, cortical structure, trabecular bone density and biomechanical comparability to human long bones [14–16].

The primary objective of the current study was to test the feasibility of DR to quantify BMD compared to DEXA and CT reference standards in an ex vivo ovine model. The secondary objectives were to quantify ovine metapodial bone density using DR through RBAE, to compare DR-derived estimates corresponding to measurements obtained from DEXA and CT, to evaluate the strength of association between DR, DEXA, and CT using correlation, regression, and reliability analyses. We hypothesize that DR-derived measurements may be associated with BMD values obtained from DEXA and CT, supporting their potential use as a reliable surrogate indicator of BMD.

## Methods

### Study design

A prospective study was conducted on 24 distal limbs obtained from 6 sheep where all metacarpal/metatarsal bones were included for imaging analysis. Determination of BMD was performed using DR examination, DEXA and CT scans.

All study procedures were done in accordance to and approved by the Institutional Animal Care and Use Committee of Faculty of Veterinary Medicine- Cairo University (Vet. CU. IACUC Approval # VetCU18042024905).

### Samples

The required sample size was determined to be 6 animals correspond to 24 examined metacarpal/metatarsal bones. Determination of sample size was based on previous studies included BMD in animal models [17] and calculation of sample size using G power software (G power 3.1.9.7, Universität Düsseldorf, Germany). Calculation was determined with the anticipation of a large effect size (f = 0.40). With an α level of 0.05 and a desired power (1-β) of 0.80. The required sample size was sufficient to detect significant differences in bone densities across different limbs (right vs. left, metacarpal vs. metatarsal) and to evaluate correlations among the three different imaging modalities.

Distal limbs were obtained from sheep that were slaughtered at the local abattoir for routine commercial purposes, no animals were euthanized specifically for this study. Inclusion criteria were skeletally mature clinically healthy sheep free from systemic disease, free from lameness, and gait abnormalities. Clinical and orthopedic examinations were performed for all sheep prior to slaughtering by two independent veterinarians. The sheep were skeletally mature male animals aging 12 ± 2 months old, weighing 55 ± 5 kg with a mean body condition score of 3 based on 5-point scale [18].

Just following slaughtering, all limbs were excised at the carpal/tarsal joints, labelled (identification number, right-left, front-hind), wrapped in plastic bags, kept at 4 °C. Imaging procedures were performed within 36 h after collection [19].

Prior to imaging, all limbs were allowed to cool at room temperature, cleaned from any mud, manure or surface contaminants. Imaging analysis was made without removal of skin or soft tissue over the examined bones.

### Determination of BMD using DEXA scan

The BMD of the metacarpal/metatarsal bones was measured using DEXA (DEXA fan beam bone densitometer, Medilink, MedixDR, Pérols, France). Prior to scanning, system calibration was made to verify scanning density using the manufacturer’s phantom.

Right and left metacarpal and metatarsal bones of each animal were imaged within the same scan. All limbs were fixed horizontally over the detector to ensure reproducible positioning of all limbs. All DEXA scans were performed within the same session using the same acquisition parameters (90 kV, 2.4 mA) using the manufacturer’s recommended standard analytic procedures.

**Fig. 1:**
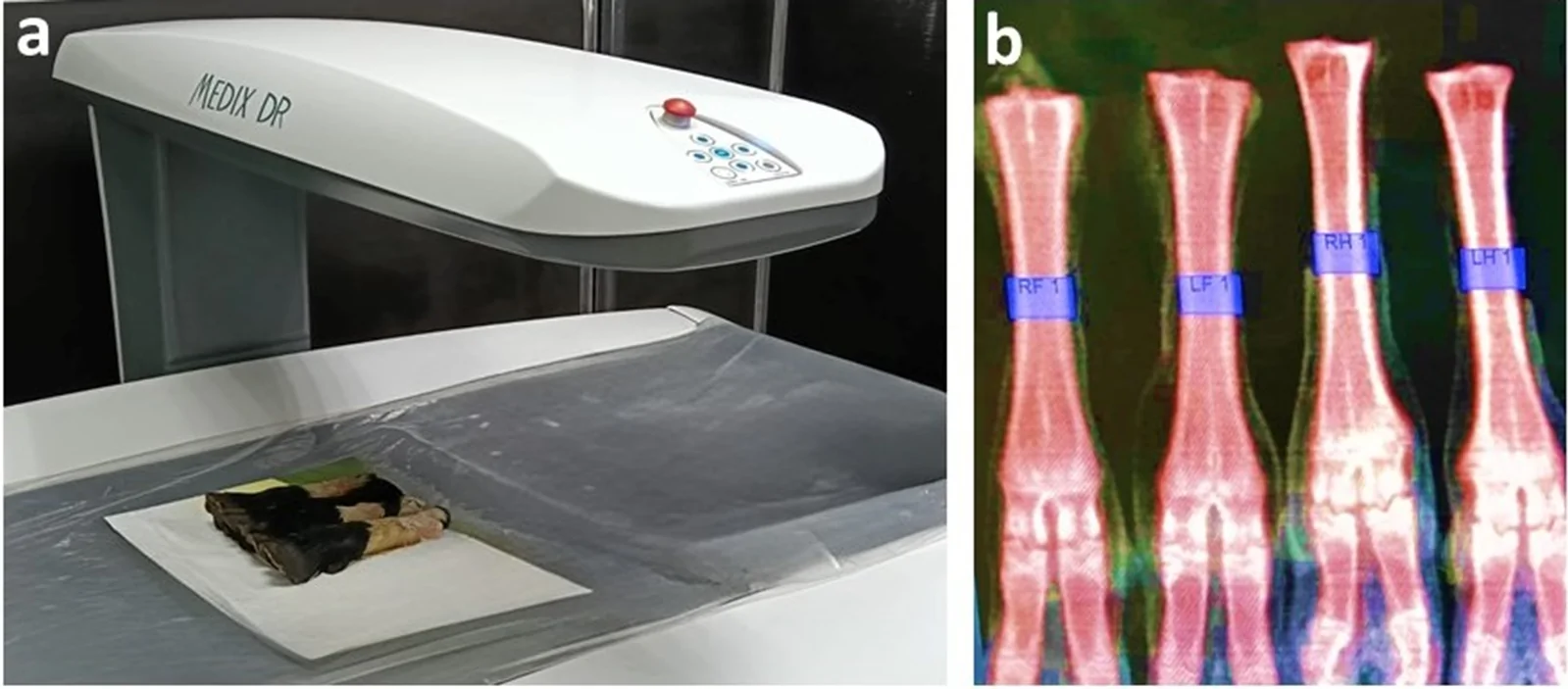
Fan-shaped densitometer for dual-energy X-ray absorptiometry (DEXA) scan (**a**). Determination of bone mineral density (BMD) was calculated for the region of interest at the mid-metacarpal and metatarsal bones of sheep (**b**)

Determination of BMD was made using the incorporated software program where the region of interest (ROI) was manually drawn at the center of each metacarpal/metatarsal bone including both cortices and the enclosed medullary cavity (Fig. 1). The ROI area was maintained across all bones to minimize variability. All measurements were performed by a single examiner to reduce inter-observer variability. Careful standardization of ROI placement was ensured to reduce measurement variability [20]. Mean BMD was expressed as gm/cm^2^.

### Determination of BMD using CT scan

**Fig. 2:**
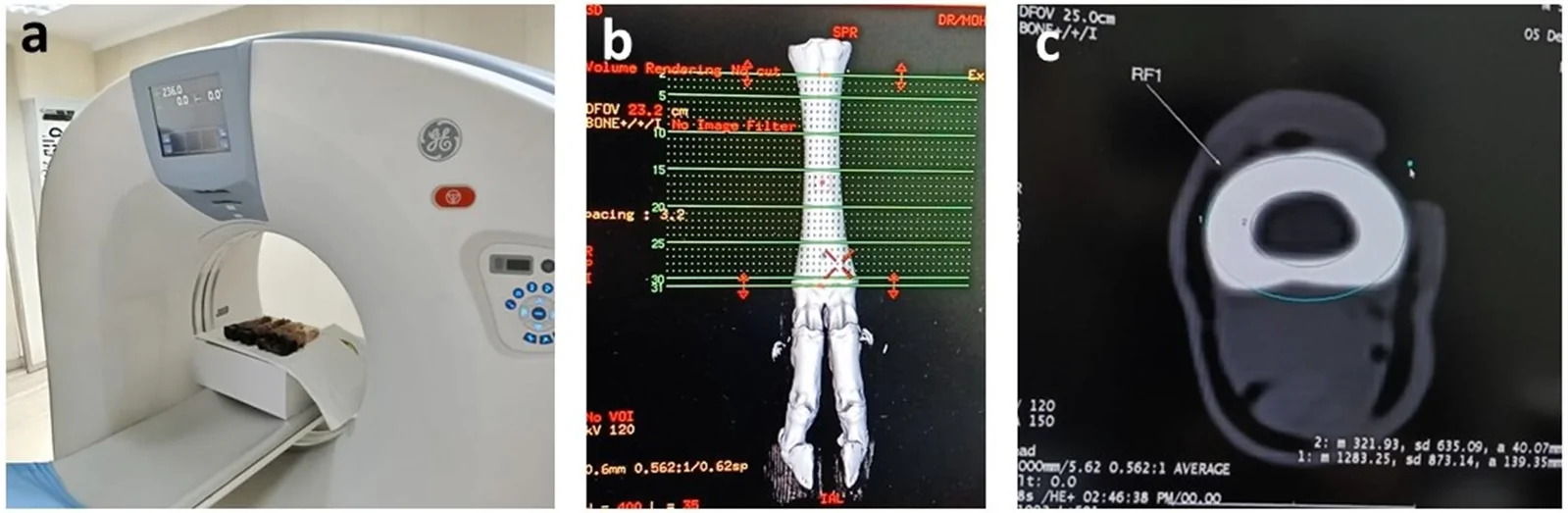
Computed tomography scan of metacarpal and metatarsal bones of sheep (**a**). Determination of bone mineral density (BMD) was calculated for the region of interest at the mid-metacarpal and metatarsal bones (**b**, **c**)

Computed tomography (CT) attenuation values (Hounsfield Units) were used as a measure of relative bone density. CT scanning was made using a multidetector row CT scanner (Optima CT520, GE Healthcare, USA). The limbs were placed horizontally parallel to the CT table plane. Investigation of the 3-D architecture of the metacarpal/metatarsal bones was made. A coronal scout was made to specify the scan length on individual bone. Then axial images were computed at a standard protocol (512 × 512 pixels, jointed slice, slice thickness 1 mm, pixel size). Limbs were scanned in 1 mm slices to measure BMD. Each metacarpal/metatarsal bone was divided into 30 segments, with the 16th segment designated as the ROI for the study (corresponds to the mid-diaphyseal region). The CT scan utilized Automatic Exposure Control (AEC) with mA modulation, incorporating 3D mA modulation featuring SmartmA and AutomA. Scanning was performed at 120 kVp and 49 mA, with 16 detector rows (Fig. 2). The degree of X-ray attenuation by the tissue being scanned was measured in Hounsfield Units (HU). As no calibration phantom was employed, these values do not represent absolute BMD but rather relative densitometric estimates.

### Determination of BMD using DR

Digital radiographic scan was obtained using (Poskom machine (PCMAX-60 H (LED), Poskom Co., Gyeonggi-do, Korea) and Varex DR flat panel detector (PaxScan^®^ 4336Wv4 Gen 2 36 × 43 cm) operated at 3.2 mA, 1 s, and 44 kVp, with a focus film distance of 90 cm.

**Fig. 3:**
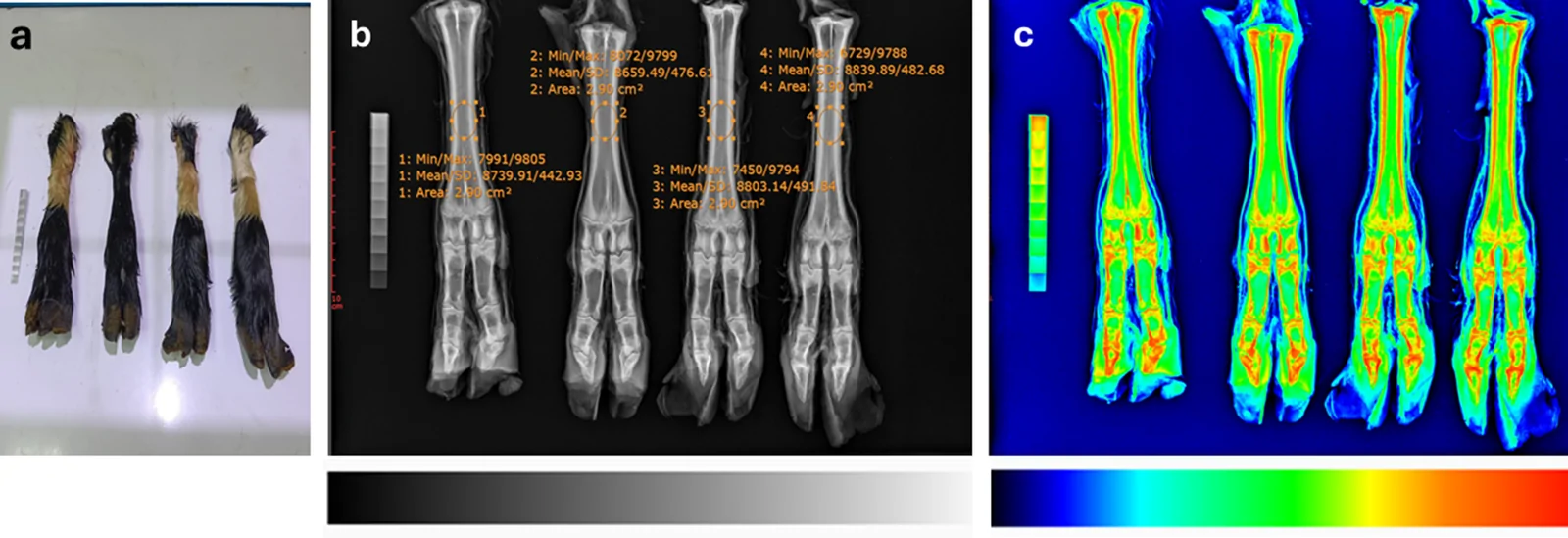
Digital radiographic examination for determination of radiographic bone density of sheep metacarpal/metatarsal bone. Placement of an aluminum step-wedge was made adjacent to bones to present findings in mmAl equivalent densities (**a**). Radiographic bone density of metacarpal and metatarsal bones was measured using a ROI that was manually selected to be in the middle of metacarpal and metatarsal bones including both medullar and cortical bone. The mean grey intensity of each pixel within the outlined region was then recorded (**b**). Radiographic bone density is represented in color scale correlated to bone radiodensity (**c**)

Radiographic imaging of the right and left metacarpal and metatarsal bones of the same animal was made while placement of an aluminum step wedges (1 mm/step,10 steps) within the same scan (Fig. 3.a). Imaging was obtained in a dorsopalmar/dorsoplantar scan, image processing was made using the incorporated software where image settings were not modified after image acquisition. The computed image processing procedure did not alter the obtained grey level values. Digital radiographs were transferred to a computer system and analyzed using DICOM viewer software (Sante DICOM viewer, SanteSoft LTD, Nicosia, Cyprus).

To enable quantification of bone density independently of post-processing and acquisition settings, the inclusion of an aluminum (step) wedge in the radiographic field-of-view has been used to be expressed as RBAE where the grey values of the bone can be expressed in millimeter aluminum equivalents (mmAl) [21]. The RBAE was utilized to use the known density of the aluminum step wedge to determine the density of the bone in aluminum equivalents, based on the optical density of the aluminum step wedge.

**Fig. 4:**
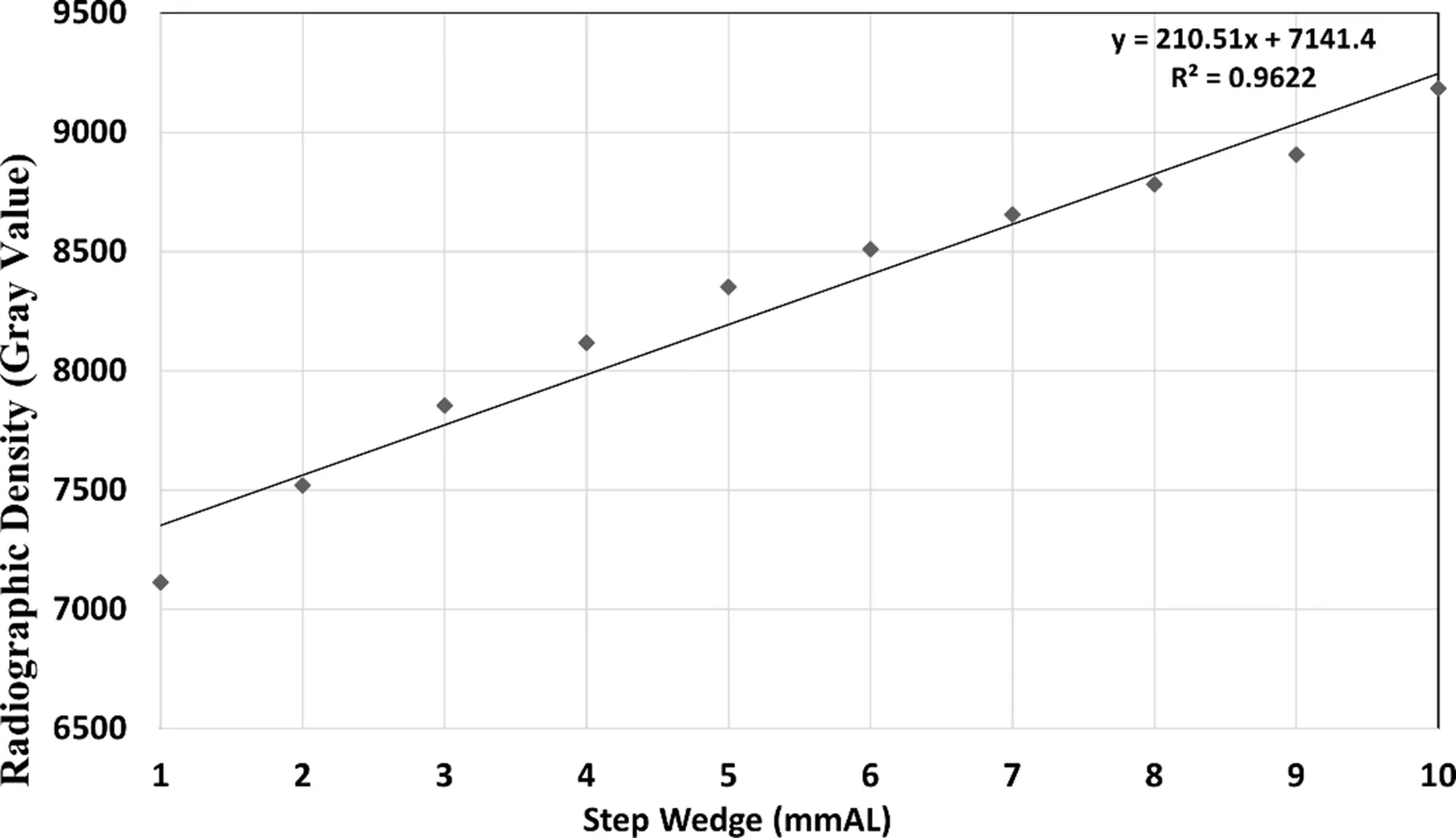
Calibration curve for radiographic quantification of bone mineral density (BMD) using an aluminum step wedge. The calibration curve was generated by plotting the mean greyscale (optical density) values obtained from each step of a standardized aluminum step wedge against the corresponding aluminum thickness (mm). Regions of interest were consistently placed over each step to minimize variability. Linear regression analysis demonstrated a strong correlation between greyscale values and aluminum thickness, validating the use of the aluminum step wedge as a reference standard. This calibration curve was subsequently used to convert radiographic greyscale values of bone regions of interest into equivalent aluminum thickness, providing a quantitative surrogate measure of bone mineral density

To create a calibration curve, the radiographic density was measured on a circular ROI on each step of the wedge (Fig. 4). Then radiographic bone density of metacarpal and metatarsal bones was measured using ROI that was manually selected to be in the middle of metacarpal and metatarsal bones including both medullar and cortical bone. The mean grey intensity of each pixel within the outlined region was then recorded (Fig. 3.b).

All image acquisitions were made in triplicates and analyses were performed by the same operator to ensure consistency.

### Statistical analysis

Data were expressed as mean ± standard deviation (SD). Normality of distribution was tested using the Shapiro-Wilk test. Within different imaging modalities, Paired t-test was conducted to detect differences among “side” (right vs. left) and “position” (front/metacarpal vs. hind/metatarsal) as within-subject factors.

To explore the relationships between imaging modalities, measurements obtained from digital radiography expressed as millimeter of aluminum equivalent (DR-mmAl), dual-energy X-ray absorptiometry expressed as gm/cm^2^ (DEXA-BMD), and computed tomography expressed as Hounsfield Units (CT-HU) were compared using Pearson correlation coefficients. Scatterplots with regression lines were generated to visualize these associations.

Because the absolute scales of measurement differed between modalities, values were standardized (z-scores) prior to cross-modality comparisons. A repeated-measures ANOVA with “modality” as the within-subject factor was then performed on the standardized data to evaluate global differences between techniques. Agreement between the three imaging modalities was assessed using both intraclass correlation coefficient (ICC) analysis and Bland-Altman plots following z-score standardization.

All statistical analyses were performed using Statistical Package for Social Sciences software (SPSS^®^ Inc. Version 25 for Windows, IBM Corporation, NY, USA). Data were considered statistically significance where the p value < 0.05.

## Results

The mean BMD for all metacarpal/metatarsal bones as obtained by dual-energy X-ray absorptiometry (DEXA-BMD) was 0.76 ± 0.09 (0.64–0.90), computed tomography (CT-HU) was 1234 ± 139 (1002–1380), and digital radiography (DR-mmAl) was 7.39 ± 0.40 (6.78–8.04).

Statistical analysis demonstrated no significant differences in bone density of the right and left metacarpal/metatarsal bones (*p* > 0.05) either by DEXA, CT, nor DR.

**Fig. 5:**
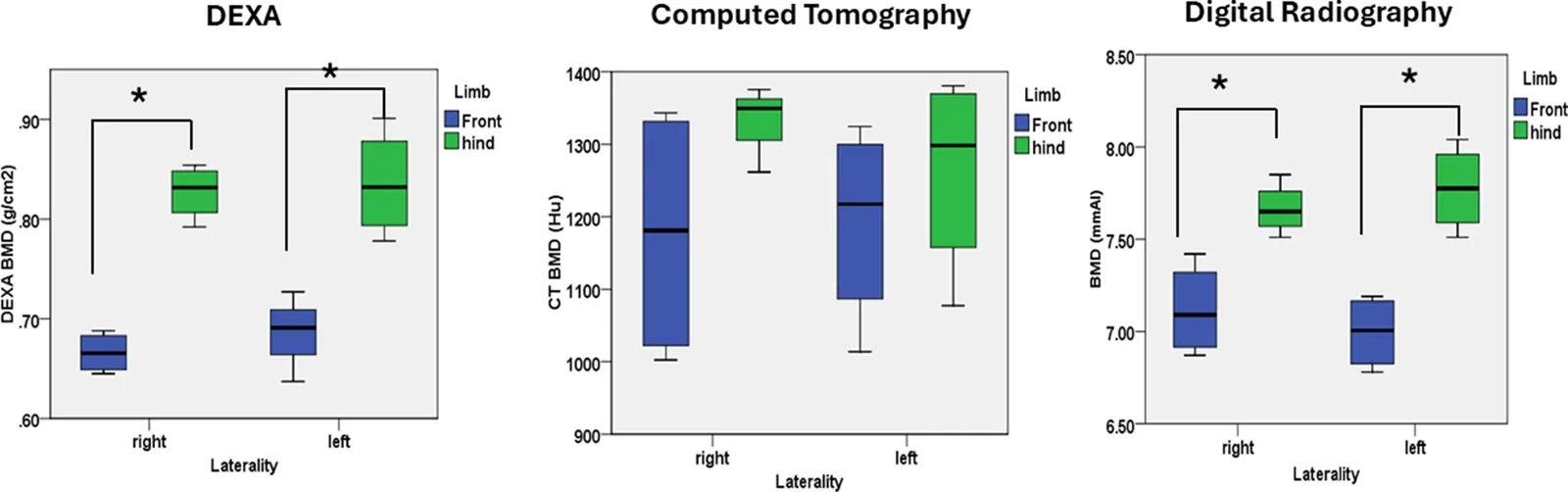
Box-and-whisker plots illustrate bone mineral density (BMD) as obtained by DEXA, computed tomography (CT), and digital radiography. For each modality, data are grouped by laterality (right vs. left) and limb (front “metacarpal” vs. hind “metatarsal”). Boxes represent the interquartile range, the central line indicates the median, and whiskers denote the minimum and maximum values. Asterisks (*) indicate statistically significant differences between metacarpal and metatarsal bones within the same laterality (*p* < 0.05)

Quantification of BMD demonstrated measurable differences between metacarpal and metatarsal bones. Similar patterns were observed in DEXA-BMD, DR-mmAl and CT-HU, where bones of the hindlimbs (metatarsal bones) consistently demonstrated higher density values than forelimbs (Fig. 5). These differences were statistically significant in DEXA-BMD (*p* = 0.000), DR-mmAl (*p* = 0.000) but not for the CT-HU (*p* = 0.226). The mean radiographic bone density of the front and hind limbs as measured by different modalities is demonstrated in Table 1.

**Table 1:** Mean radiographic bone density as obtained by DEXA, CT, and digital radiography of the right and left, front and hind bones

		DEXA (g/cm²)	CT (Hu)	Digital Radiography mm aluminum equivalent (mmAl)	
**Laterality**	Right	0.76 ± 0.09	1242 ± 154	7.39 ± 0.35	
	Left	0.77 ± 0.09	1250 ± 128	7.38 ± 0.46	
	**P value**	0.522	0.700	0.956	
**Limb**	Front (metacarpal)	0.67^a^ ± 0.03	1209 ± 141	7.05^a^ ± 0.22	
	Hind (metatarsal)	0.83^b^ ± 0.36	1291 ± 109	7.72^b^ ± 0.19	
	**P value**	0.000	0.226	0.000	

Different superscript letters indicate statistically significant differences within the same column

Cross-modality analysis demonstrated strong positive correlations between DEXA-BMD and DR-mmAl (*r* = 0.779, *p* = 0.001), moderate positive correlation between DEXA-BMD and CT-HU and (*r* = 0.520, *p* = 0.05), and CT-HU and DR-mmAl (*r* = 0.536, *p* = 0.04). These results indicate that despite differences in measurement scales, the three imaging modalities provide broadly consistent ranking of bone density across different bones (Table 2).

**Table 2:** Correlation analysis between bone mineral density (BMD) measurements as obtained by DEXA (DEXA-derived bone mineral density (DEXA-BMD)), digital radiography-derived BMD expressed as equivalent aluminum thickness (DR-mm Al) and computed tomography (CT) attenuation values expressed in Hounsfield units (CT-HU)

Variable Pair	*r*	95% Confidence of Interval		*p*-value
		Lower bound	Upper bound	
**DEXA-BMD vs. DR-mmAl**	0.799	0.43	0.93	0.001
**DEXA-BMD vs. CT-HU**	0.520	-0.02	0.83	0.050
**CT-Hu vs. DR-mmAl**	0.536	0.04	0.82	0.040

Analysis of the standardized (z-scores) across modalities demonstrated that in Mauchly’s test the assumption of sphericity was not violated for the within-subjects factor modality (W = 0.803, χ²(2) = 2.631, *p* = 0.268). Therefore, the uncorrected degrees of freedom were used. Repeated measures ANOVA revealed that the effect of “modality” on the outcome measure was not statistically significant, F (2, 26) = 0.178, *p* = 0.838, indicating that the measured values did not differ across modalities. Analysis of laterality (right vs. left) similarly showed no significant differences indicating no systematic bias was detected between modalities after normalization. The mean value for the right side was − 0.081 (95% CI: -0.782 to 0.620), while the left side mean was 0.178 (95% CI: -0.523 to 0.879). Pairwise comparisons confirmed the absence of significant differences between sides (mean difference = -0.259, SE = 0.455, *p* = 0.580). The univariate test of laterality also yielded a non-significant effect, F(1, 12) = 0.324, *p* = 0.580. Similarly, comparisons between front and hind limbs showed no significant differences. The mean value for front limbs was − 0.222 (95% CI: -0.923 to 0.479), while hind limbs had a mean of 0.319 (95% CI: -0.382 to 1.020). Pairwise comparisons indicated a non-significant difference (mean difference = -0.541, SE = 0.455, *p* = 0.256), and the univariate test confirmed no main effect of limb position, F (1, 12) = 1.416, *p* = 0.256. Taken together, these analyses demonstrate that quantification of bone density did not differ significantly between the three modalities, between right and left sides, or between front and hind limbs.

Reliability analysis using ICC analysis and two-way mixed-effects model with absolute agreement demonstrated moderate to good reliability for single modality (ICC = 0.750, 95% CI: 0.538–0.767, *p* < 0.001) and good reliability for average modalities (ICC = 0.801, 95% CI: 0.605–0.908, *p* < 0.001) indicating increased stability when modalities were considered collectively.

**Fig. 6:**
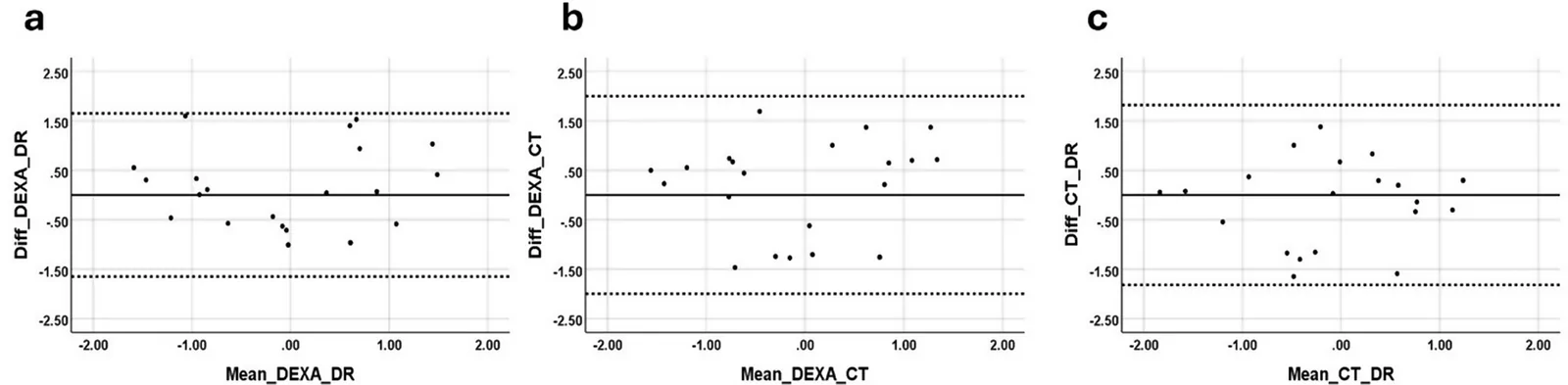
Bland-Altman analysis used to assess agreement between the three imaging modalities following z-score standardization. Across all comparisons, the mean differences were approximately zero, indicating no evidence of systematic bias between methods. The narrowest limits of agreement were observed between DEXA and DR (**a**) (-1.65 to 1.65), indicating the strongest agreement. Agreement between DEXA and CT (**b**) was the widest limit (-2.0 to 2.0), while the moderate agreement was found between CT and DR (**c**) (-1.82 to 1.82)

Bland-Altman analysis demonstrated no systematic bias between modalities, as the mean differences were approximately zero across all comparisons. The narrowest limits of agreement were observed between DEXA and DR (-1.65 to 1.65), indicating the strongest agreement. Agreement between DEXA and CT was the widest limit (-2.0 to 2.0), while the moderate agreement was found between CT and DR (-1.82 to 1.82). Visual inspection of the Bland-Altman plots did not reveal any clear proportional bias, with differences appearing randomly distributed across the range of measurements (Fig. 6).

## Discussion

The present study demonstrated that digital radiography, when calibrated and expressed as RBAE values, can provide quantitative measurements that is correlated with BMD values obtained from the DEXA gold-standard modality. The results supported the feasibility of using DR as a practical surrogate method for bone density assessment in long bones, which will be helpful particularly in circumstances where advanced imaging equipment may not be readily available.

Consistent with previous literature in equine [22, 23], DEXA showed high precision and reproducibility in measuring BMD of ovine metacarpal and metatarsal bones. In the present study, CT measurements were obtained as HU values without the use of a calibration phantom; therefore, they were treated as relative densitometric measurements rather than absolute BMD. The primary aim of including CT was to provide a comparative reference reflecting relative differences in bone density across samples, rather than to perform quantitative CT (QCT).

The strong correlations observed between DR and DEXA, moderate positive correlation between DR and CT indicate that radiographic greyscale values, when properly calibrated, reflect mineral content with acceptable accuracy.

The moderate positive correlations observed between CT-HU and DEXA-BMD values (*r* = 0.520, *p* = 0.05) and between CT-HU and DR-mmAl measurements (*r* = 0.536, *p* = 0.04) suggesting that while CT provides structural and density-related information that aligns reasonably well with mineral density estimates, it also reflects additional tissue characteristics- such as trabecular architecture, marrow composition, and cortical thickness, that may introduce variability relative to purely mineral-based measurements. Similarly, the correlation between CT-HU and DR-mmAl values confirms that the radiographic quantification method captures mineral-associated density changes detectable by CT, further supporting its biological and mechanical relevance. Conversely, DR’s two-dimensional nature inherently limits precise separation of cortical and trabecular contributions, which may explain minor discrepancies observed during agreement analysis. Correlation and regression analyses demonstrated a significant association between DR and both DEXA and CT, indicating that DR-mmAl values are reflective of underlying variations in BMD.

The use of RBAE expressed as DR-mmAl is a projection-based radiographic method, providing an attenuation-derived estimate of bone mineral content that is conceptually closer to areal BMD measurements such as DEXA than to true volumetric density obtained from CT, and it is inherently influenced to some extent by bone geometry and size, a characteristic shared by all two-dimensional densitometric techniques.

Reliability testing using ICC analysis indicated that while individual measurements of DEXA-BMD, CT-HU, DR-mmAl show moderate to good consistency, averaging multiple modalities substantially improves reliability, yielding good agreement. The use of an absolute agreement definition further indicated that this reliability reflected not only consistency in ranking but also similarity in absolute values across measurements. This suggests that DR is capable of providing reproducible estimates that track changes in bone density.

Bland-Altman analysis further demonstrated no systematic bias between modalities following standardization, with mean differences approximating zero. However, the limits of agreement varied across modality pairs, indicating the presence of measurement variability and reinforcing that the techniques are not directly interchangeable. Collectively, these findings demonstrate sufficient association and reliability of DR to serve as a practical surrogate indicator of BMD in an experimental context. In particular, DR appears suitable for detecting relative differences, trends, and group-level variations, supporting its use as a low-cost and accessible tool in bone research applications.

The variation in the magnitude of the observed associations between DR and DEXA and CT likely reflects inherent methodological differences between projection-based and volumetric imaging techniques, rather than a limitation of DR alone; in particular, the closer structural similarity between DR and DEXA (both being projectional measures of bone mineral content) may explain the stronger correlation observed with DEXA, whereas CT provides true three-dimensional volumetric density measurements that are not directly comparable to projection-derived estimates.

Designing the study as an ex vivo model was intended to minimize sources of variability due to inconsistent limb positioning, and motion artefacts. This allowed reliable standardization of radiographic exposure settings, beam angle, and object-detector distance, factors known to significantly influence radiographic greyscale values [24].

The use of metacarpal and metatarsal bones has been used as ideal sites for quantifying bone mineral density in quadrupedal species [22, 23, 25–28]. The metacarpal and metatarsal bones have a relatively uniform geometry, and crucial biomechanical function. As primary weight-bearing long bones, they are subjected to predictable mechanical loading forces that directly influence mineralization through the principles of Wolff’s law [23]. Their cortical structure provides a stable and reproducible region for evaluating density-related changes, while their accessibility facilitates high-quality imaging with minimal soft-tissue interference [11].

Setting and standardization of an anatomical landmark is particularly crucial when measuring BMD in order to achieve reproducible and comparable measurements [11, 29]. The region of interest was selected at mid-shaft region of the metacarpal and metatarsal bones to provide an optimal and highly reproducible site for quantitative assessment of bone mineral density [26–28]. The mid-shaft is easily visualized with minimal soft-tissue superimposition, facilitating consistent ROI placement across imaging modalities. This diaphyseal segment is predominantly composed of dense cortical bone, which offers greater structural uniformity and less anatomical variation compared with metaphyseal or epiphyseal regions [30]. In addition, BMD measurements obtained from the mid-shaft are less affected by trabecular complexity, growth-plate remnants, or joint-related remodeling, thereby enhancing measurement precision and reducing biological noise [11, 30]. Biomechanically, the diaphysis is the primary site of bending and torsional stresses during locomotion, meaning that mid-shaft BMD accurately reflects adaptive responses to habitual mechanical loading. These characteristics make the mid-shaft ROI a robust, standardized, and biologically meaningful location for evaluating BMD in experimental and clinical studies involving metacarpal and metatarsal bones.

The use of an aluminum step wedge with multiple steps of known and progressively increasing thicknesses provided a calibrated reference greyscale-density curve for each radiograph. This internal calibration compensates for variations in exposure parameters, beam quality, object thickness, and detector sensitivity, thereby enhancing the accuracy and reproducibility of radiographic BMD measurements. This calibration allowed transformation of radiographic image into a semi-quantitative modality capable of detecting relevant differences in mineral density [27].

Data obtained from the current study revealed that across all three imaging modalities, a consistent pattern had emerged where the metatarsal bones (hindlimbs) exhibited significantly higher BMD values than the metatarsal bones (forelimbs), irrespective of laterality. Similar reports were previously reported in Thoroughbred horses [11].

The higher BMD recorded in metatarsal bones could be attributable to their greater biomechanical workload. Quadrupedal species rely heavily on their hindlimbs for propulsion, acceleration, deceleration, and stabilization during movement [31]. This results in increased mechanical loading and cyclic strain compared with forelimbs. According to Wolff’s law, repeated mechanical stress stimulates osteoblastic activity and enhances mineral deposition, ultimately promoting stronger and more mineral-dense bone [32]. The current results align well with these principles and add quantitative support to the long-standing knowledge regarding functional bone adaptation in weight-bearing limbs.

Comparing the right and left limbs, no significant laterality differences were detected within forelimbs or hindlimbs, suggesting symmetrical loading patterns and balanced mechanical function between right and left sides. This result aligned with the previously reported results in equine metacarpal bones and phalanges [11, 33, 34]. The finding also provides useful baseline information for future studies investigating unilateral orthopedic disease, developmental asymmetries, or limb-specific interventions. On the other hand, a human study revealed that hand preferences may result in differences in BMC due to greater bone size and area but without significant differences in BMD between bilateral forearm bones [29].

Findings obtained from the current study contribute valuable reference data for normal bone mineral density in ovine limbs and highlight the strong influence of functional biomechanics on skeletal adaptation. These insights may help to inform future research on metabolic bone disease, orthopedic rehabilitation, limb biomechanics, and the validation of imaging-based BMD assessment tools.

The results of the present study underscore the potential utility of digital radiography (DR) as an accessible and cost-effective tool for preliminary assessment of bone mineral density. DR is widely available, rapid, and straightforward to perform, typically requiring only brief exposure times and no general anesthesia. Its relatively low capital investment further supports its implementation in routine clinical and veterinary settings, reinforcing the concept of opportunistic bone health assessment [20, 35]. In this context, DR may enable simultaneous diagnostic evaluation and quantitative estimation of bone density within a single examination, thereby reducing examination time, cost, and overall radiation exposure.

In conclusion, DR could serve as a rapid method for baseline BMD characterization prior to orthopedic or regenerative experiments, selection of uniform experimental cohorts, or long-term monitoring of bone healing and remodeling when advanced imaging is unavailable.

Despite the robust findings demonstrated, several limitations should be acknowledged. First, we acknowledge the relatively small sample size and the use of a limited number of animals, which may restrict statistical power and generalizability. Relatedly, the inclusion of multiple limbs from the same animal introduces potential intra-subject correlation, which we have addressed analytically and now explicitly discuss as a potential source of bias. Second, the study was conducted in an ex vivo ovine model under controlled conditions. While this design allows for precise, standardized imaging across modalities, it does not fully replicate in vivo clinical settings, where factors such as soft tissue variability, motion, and patient heterogeneity may influence measurements. As such, the translational applicability of the findings should be interpreted with caution.

Third, the biological diversity of the samples is limited, as the specimens were relatively homogeneous in terms of age, health status, and skeletal characteristics. This was intentional for a feasibility study but may limit the applicability of the results to broader and more heterogeneous populations. Fourth, CT measurements were based on attenuation values expressed in HU without the use of a calibration phantom. As a result, CT data represents relative densitometric measurements rather than absolute BMD, which may limit direct comparability with DEXA-BMD values. Fifth, the use of z-score standardization, while necessary to allow comparison across modalities with different measurement scales, may have obscured potential systematic differences between methods and limits direct clinical interpretation of agreement in absolute units. Sixth, the measurements of each scan were performed by a single examiner. Although this approach minimized inter-observer variability and ensured methodological consistency, it may have introduced observer bias and precluded assessment of inter-observer reproducibility. Future studies involving multiple independent examiners and evaluation of inter- and intra-observer reliability would further strengthen the reproducibility and generalizability of the findings. Finally, we acknowledge that the study is primarily focused on feasibility and methodological validation rather than clinical outcome assessment. Accordingly, while the findings are encouraging, further investigations involving larger and more diverse cohorts, as well as in vivo study designs, are required to establish the clinical utility of the proposed RBAE approach.

## Conclusion

Overall, the results of this study primarily support the role of DR as a surrogate indicator of BMD rather than a direct substitute for established imaging modalities. Further studies incorporating larger sample sizes, calibrated CT protocols, and in vivo validation are warranted to confirm and extend these findings. In addition, future work should evaluate the sensitivity of DR-based approaches to detect longitudinal changes in BMD associated with disease progression or therapeutic interventions.

## Data Availability

The data that support the findings of this study are available from the corresponding author upon reasonable request.
